# Institut Georges Lopez-2M as a Novel Lung Preservation Solution Attenuates Ischemia-Reperfusion Injury in a Rat *Ex Vivo* Lung Perfusion Model 

**DOI:** 10.3389/ti.2026.15993

**Published:** 2026-02-19

**Authors:** Annalisa Barbarossa, Jan Van Slambrouck, Cara Trivett, Phéline Kortleven, Cedric Vanluyten, Alberto Martin Medina, Xin Jin, Nicole Jannis, Balin Özsoy, Sandra Claes, Dominique Schols, Tine Wylin, Karen Moermans, Steve Stegen, Arnau Panisello Rosello, Ilhan Inci, Paul De Leyn, Bart Vanaudenaerde, Jacques Pirenne, Elizabeth A. V. Jones, Laurens J. Ceulemans

**Affiliations:** 1 Laboratory for Respiratory Diseases and Thoracic Surgery, Katholieke Universiteit Leuven, Leuven, Belgium; 2 Department of Thoracic Surgery, University Hospitals Leuven, Leuven, Belgium; 3 Centrum voor Moleculaire en Vasculaire Biologie, Katholieke Universiteit Leuven, Leuven, Belgium; 4 Laboratory of Molecular Virology and Gene Therapy, Katholieke Universiteit Leuven, Leuven, Belgium; 5 Department of Microbiology, Immunology and Transplantation, Rega Institute for Medical Research, Molecular, Structural and Translational Virology Research Group, Katholieke Universiteit Leuven, Leuven, Belgium; 6 Laboratory of Abdominal Transplantation, Transplantation Research Group, Department of Microbiology, Immunology and Transplantation, Katholieke Universiteit Leuven, Leuven, Belgium; 7 Clinical and Experimental Endocrinology, Department of Chronic Diseases and Metabolism, Katholieke Universiteit Leuven, Leuven, Belgium; 8 Translational Research and Innovation in Surgery Group (GITIC), Department of General and Digestive Surgery, La Paz University Hospital - IdiPaz, Madrid, Spain; 9 Klinik Hirslanden Zurich, Thoracic Surgery Clinic, Zurich, Switzerland; 10 Universiteit Maastricht Cardiovascular Research Institute Maastricht, Maastricht, Netherlands

**Keywords:** endothelial glycocalyx, ischemia-reperfusion injury, lung preservation, polyethylene glycol (PEG), preservation solution

## Abstract

*Institut* *Georges Lopez-2M* (*IGL-2M*), a novel preservation solution containing polyethylene glycol (PEG 35kD, 5 g/L), preserves mitochondrial integrity and redox balance in liver grafts. This study assesses *IGL-2M*’s effect on lung preservation during prolonged cold ischemia. Rat’s heart-lung blocks were procured and subjected to 18 h cold ischemia (4 °C). Lungs were flushed and preserved using one of these preservation solutions: *OCS*, *Perfadex Plus*, *IGL-2M* (n = 6/group). Following ischemia, lungs underwent up to 7 h normothermic *ex vivo* lung perfusion. Edema was quantified by weight gain. Lung physiological parameters were recorded. Perfusate, bronchoalveolar lavage (BAL), and tissue samples were collected. All lungs in *IGL-2M* group completed 7 h EVLP protocol. Compared to *OCS*, *IGL-2M* reduced edema formation (p < 0.01), preserved superior compliance (p < 0.01), and maintained lower pulmonary vascular resistance (p < 0.01). *IGL-2M* showed lower perfusate concentrations of IL-1β (p < 0.05), IL-6 (p < 0.05), and TNF-α (p = 0.08). In BAL, *IGL-2M* reduced IL-1β (p < 0.01), IL-6 (p < 0.05), TNF-α (p < 0.01), and CXCL1 (p < 0.01). *IGL-2M* showed lower release of Syndecan-1 (p < 0.05). Compared to *Perfadex Plus*, *IGL-2M* was not inferior, with reduced expression of TNF-α in the perfusate (p < 0.05). *IGL-2M* effectively prevents edema development like *Perfadex Plus*. *IGL-2M* results in decreased inflammation and a stronger endothelial lining, making it a promising solution for lung preservation.

## Graphical Abstract

**Figure:**
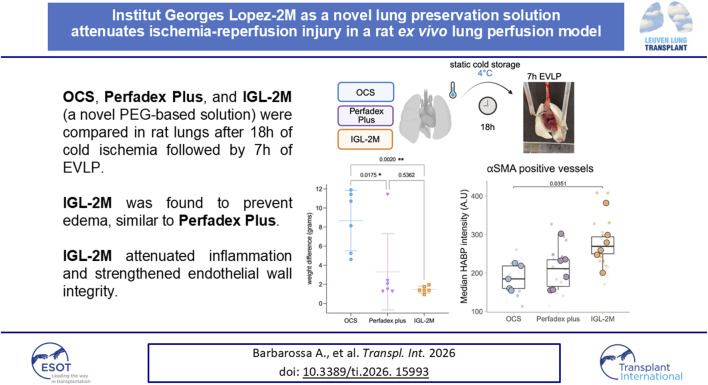

## Introduction

Ischemia–reperfusion injury (IRI) is one of the key challenges in lung transplantation and plays a pivotal role in the development of primary graft dysfunction (PGD), leading to early morbidity and mortality [1]. The sequence of ischemia during organ procurement, static cold storage, and subsequent reperfusion after implantation triggers a cascade of cellular and molecular events that culminate in tissue injury [2]. At the onset of ischemia, an imbalance between metabolic supply and demand disrupts epithelial and endothelial homeostasis, alters mitochondrial function, and induces ionic disequilibrium. Cold preservation further amplifies this process through the generation of reactive oxygen species (ROS), inflammatory cytokine release, microvascular injury, and epithelial barrier disruption, ultimately resulting in lung edema and PGD following reperfusion [3, 4].

A central element in IRI is the inflammatory response, which is amplified upon reperfusion. Pro-inflammatory cytokines such as IL-1β, IL-6 and TNF-α, play a key role by activating endothelial cells, promoting leukocyte adhesion, and increasing vascular permeability [5, 6]. Chemokines like CXCL1 further drive neutrophil recruitment, while dysregulation of anti-inflammatory mediators such as IL-10 contributes to an imbalance between pro- and anti-inflammatory signaling [7, 8]. This cytokine storm accelerates endothelial dysfunction, microcirculatory failure, and edema formation, ultimately impairing graft function, leading to PGD [9].

Progress in optimizing lung preservation remains limited. Current preservation solutions differ across centers but share common design principles: colloids to counteract cellular edema, buffers to stabilize pH, antioxidants to scavenge ROS, and metabolic precursors to support ATP regeneration [10, 11]. However, clinical outcomes indicate that existing solutions provide partial protection, and novel approaches are needed to better preserve vascular integrity and attenuate IRI, especially in the context of evolving lung preservation practices toward controlled hypothermic storage and prolonged preservation time [12].

One promising strategy involves the use of polyethylene glycol (PEG)-based preservation solutions. *Institut Georges Lopez-2* (*IGL-2®*), with PEG 35 kDa at 5 g/L, has been shown to stabilize mitochondrial function, preserve redox balance, and reduce endothelial injury in experimental liver transplantation [13, 14].

The aim of this study is to evaluate whether a further modified PEG-based preservation solution *IGL-2M* improves lung preservation during prolonged cold ischemia compared to clinically used Organ Care System lung solution (*OCS)* and *Perfadex Plus*. The primary endpoint of the study is to assess the success rates of 7 h of EVLP and edema formation. We analyzed the effect on edema, inflammation, and endothelial injury, using a rat *ex vivo* lung perfusion (EVLP) model to mimic IRI.

## Materials and Methods

### Animals

Male Sprague-Dawley rats (350–400 g, Janvier Labs, France) received adequate care, and the study was performed after authorization by local Ethical Committee for Animal Experimentation (Ethische Commissie Dierproeven) (P128/2023).

### Surgical Techniques

Rats were anesthetized with 5% isoflurane and maintained with 3% isoflurane during lung procurement. A tracheostomy was performed, followed by mechanical ventilation with rodent ventilator (R415VentStarSmallAnimalVentilator; RWD Life Science Co., Ltd. Guangdong, P.R. China). A tidal volume (TV) of 8 mL/kg, ventilatory rate (VR) of 70/min, positive end-expiratory pressure (PEEP) of 3cmH_2_O, and a fraction of inspired oxygen (FiO_2_) of 50% were applied. A laparotomy was performed, and 200U/kg heparin (LeoPharma, Denmark) was injected into abdominal caval vein followed by sternotomy with removal of thymus to gain access to the pulmonary artery (PA). Exsanguination was obtained by cutting abdominal caval vein. The PA (inflow) was cannulated through an incision in the right ventricular outflow tract, while left atrium (outflow) was cannulated through an incision in the apex of left ventricle and after dilatation of mitral valve. Cold pulmonary antegrade flush was performed using one of three preservation solutions (20cc at 4 °C): *OCS*, *Perfadex Plus*, *IGL-2M* and 500U heparin. After pulmonary flush, pneumonectomy was performed. Trachea was clamped at inspiration and transected. The heart-lung block was weighed, submerged in same cold preservation solution used for the flush, and stored for 18 h in a temperature-controlled cold room at 4 °C. A cold ischemic time of 18 h was chosen to establish a severe, robust and reproducible injury model that mimics ischemia reperfusion injury, thereby enabling discrimination between preservation solutions under extreme conditions, which would not be achievable with shorter cold ischemia times.

### *Ex-Vivo* Lung Perfusion

Lungs were mounted on an isolated perfused lung system for rats (IPL-2 platform; Hugo-Sachs Elektronik, Germany). The *ex vivo* lung perfusion protocol used was inspired by the protocols employed in the research laboratories of Lausanne and Zurich [15, 16]. A 75 mL of acellular albumin-rich Steen solution (XVIVO, Sweden) at 7 °C was used as perfusate supplemented with 1 mL of diluted Tham Koler 3M. Flow-controlled perfusion was started at 1% of cardiac output (CO) and was increased to 7.5% CO at incremental steps within 20 min. Perfusate temperature was increased to 37 °C within 25 min. Volume-controlled ventilation was started after 20 min reperfusion time at a 3 mL/kg TV, 10/min VR, 21% FiO_2_ and 3cmH_2_O PEEP. TV was increased to 6 mL/kg and VR to 30/min after 30min. The perfusate was continuously deoxygenated through a membrane filter (D150, Medica, Italy) with a 12% CO_2_/6% O_2_/82% N_2_ sweep gas. A recruitment maneuver with a TV of 10 mL/kg was performed every hour, followed by 3min of ventilation at 100% FiO_2_ and subsequent gas analysis of inflow and outflow perfusate samples (ABL800 FLEX, Radiometer, Denmark). Differential perfusate pO_2_ (outflow-inflow) (ΔpO_2_) and outflow perfusate lactate levels were recorded.

Respiratory physiology data were measured and recorded using dedicated software (Pulmodyn HSE software, Germany). Pulmonary arterial pressure (PAP), pulmonary vascular resistance (PVR), and compliance were monitored. Left atrium pressure was set at 2-3 cmH2O. Hourly outflow perfusate samples (1 mL) were collected, immediately snap frozen in liquid nitrogen and stored at −80 °C.

**FIGURE 1:**
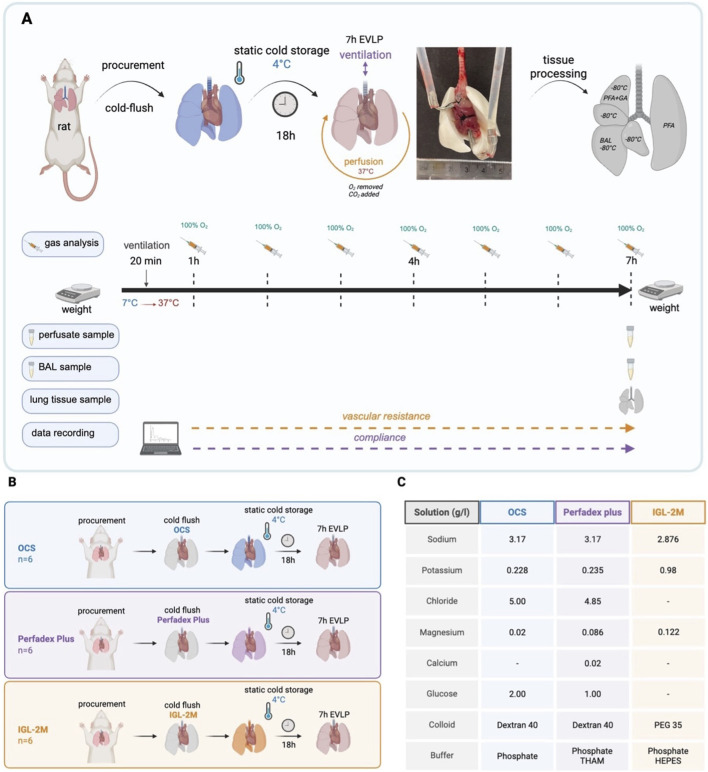
**(A)** Experimental protocol and *ex vivo* lung perfusion sampling. **(B)** Study groups; **(C)** Composition of preservation solutions. Information about *OCS* solution’s composition was found on the clinic bag that held the solution. Details about *Perfadex Plus* were found on XVIVOgroup website, and information about *IGL-2M* was provided by IGL-company. Created in https://BioRender.com.

EVLP protocol was continued for a total of 7h, unless massive edema impeding further ventilation forced a premature stop to prevent fluid from reaching the trachea, afterwards heart-lung block was weighed. Prolonged EVLP (7 h) was chosen to allow clear detection of inflammatory differences between groups. An EVLP model to mimic reperfusion, rather than a transplant model, was used to reduce animals required. Bronchoalveolar lavage (BAL) samples of 1 mL 0.9% NaCl were collected from right lower lobe and snap-frozen in liquid nitrogen. Left lobe was instilled and fixed with 4% formaldehyde (Avantor, U.S). Remaining lobes were snap-frozen in liquid nitrogen and stored at −80 °C (Figure 1A).

### Study Groups and Preservation Solutions

Cold pulmonary antegrade flush and static preservation were performed using one of three preservation solutions: *OCS* lung solution (TransMedics), *Perfadex Plus* (XVIVO), or *IGL-2M* (*Institut Georges Lopez 2 modified*) after unblinded randomization.

For each group (n = 6/group), the lungs were flushed with 20cc sterile preservation solution, pre-cooled to 4 °C, via pulmonary artery during procurement. Lungs were then preserved in the specific preservation solution at 4 °C for 18 h. After the preservation phase, EVLP was performed (Figure 1B). During EVLP, Steen solution (XVIVO, Sweden) was used as perfusate for all groups.

*OCS* lung solution is an FDA-cleared extracellular, low-potassium solution based on colloid Dextran 40 (50 g/L). It is used in clinics for normothermic perfusion and in our center for static cold preservation of donor lungs.

*Perfadex Plus* is a ready-to-use extracellular colloid-based (Dextran 40, 50 g/L) and is used by most lung transplant centers for cold flush and static preservation. Unlike *OCS*, *Perfadex Plus* is pre-buffered and pre-supplemented with calcium ions and THAM.

*IGL-2M* is a novel polyethylene glycol (PEG-35kDa) based extracellular preservation solution, representing a modified version of IGL-1 and IGL-2 with improved antioxidant and anti-inflammatory properties. Originally designed to offer protection for steatotic livers [17]. It has not been used in clinic (Figure 1C)

### Perfusate Samples, Bronchoalveolar Lavage Samples, and Lung Tissue Analysis

For analysis of cytokines, a 50 μL volume of either perfusates collected at 7 h EVLP and BAL was assayed using Rat Procarta 6-plex Luminex assay (Thermo Fisher Scientific).

RNA was isolated from frozen tissue biopsies. 25mg of frozen tissue RNA from the upper, middle, lower, and accessory lobes was combined with a 3 mm tungsten carbide bead (Qiagen, Ref#69997) and 300 µL of Aurum™ RNA lysis buffer (Bio-Rad, Ref#7326820), then homogenized using a TissueLyser II (Qiagen). RNA extraction was performed with TRIzol™ Reagent (Thermo Fisher Scientific, Ref#15596026) following the manufacturer’s protocol, and the RNA phase was further purified using the Aurum™ Total RNA Mini Kit (Bio-Rad, Ref#7326820). The RNA concentration and purity were assessed using NanoDrop oneC (Thermo Fisher Scientific). c-DNA was synthesized from 200 ng RNA using Moloney Murine Leukemia Virus Reverse Transcriptase (M-MLV, Life Technologies, CA, USA). Next, the real-time qPCR reaction was performed on a LightCycler 96W system (Roche Diagnostics, Vilvoorde, Belgium) with Taqman Fast Universal PCR Master Mix and Taqman Gene Expression Assays IL-1β (Gene expression assay: Rn00580432_m1), IL-6 (Gene expression assay: Rn01410330_m1), TNF-α (Gene expression assay: Rn00562055_m1), NFKB (Gene expression assay: Rn01399572_m1), VCAM-1 (Gene expression assay: Rn00563627_m1), Bcl2 (Gene expression assay: Rn99999125_m1), Bcl2l1 (Gene expression assay: Rn00437783_m1), bax (Gene expression assay: Rn01480160_g1) and TaqMan^®^ Fast Universal PCR Master Mix (Applied Biosystems^®^, Life Technologies, CA, USA). Thermocycling conditions consisted of an initial denaturation of 60s at 95 °C, followed by 45 cycles of 95 °C for 5s and 60 °C for 30s. Quantification of input target amount was analyzed by cycle threshold (Ct) value, the point at which the sample PCR amplification plot crosses the threshold. All data were normalized to GAPDH (Gene expression assay: Rn00562055_m1), and differences in gene expression were calculated as dCt values.

### Lung Tissue Staining for Hyaluronan-Binding Protein

Paraffin-embedded sections were used for hyaluronic acid staining using a biotinylated hyaluronan binding protein (Sigma, 385911-50UG). Sections were baked at 65 °C and allowed to cool completely. Deparaffinization and dehydration were performed by sequentially immersing slides in xylene followed by a reducing ethanol gradient (100%, 95%, 70%, 50%). Slides were then washed once with distilled water before performing antigen retrieval (Target Retrieval Solution, Dako). Following incubation with protein blocking solution (TNB protein blocking solution) and endogenous biotin blocking (Biolegend) samples were incubated in solution containing hyaluronan binding protein (1/100) and αSMA-Cy3 antibody (1/400, Sigma, C6198) overnight with gentle rocking. Slides were washed three times with TNT Buffer (0.1M Tris.HCl (pH 7.5), 0.15M NaCl, 0.05% Tween®-20). To detect bound hyaluronan, slides were incubated in the dark with streptavidin conjugated to Alexa488 (1/400, Thermo Fisher Scientific) for 1 h. Slides were washed three times in TNT, counterstained with DAPI and mounted with ProLong™ Gold Antifade Mountant (Thermo Fisher Scientific). All steps were performed at room temperature.

Fluorescent images were taken with a Zeiss 700 M confocal microscope (×20 magnification). Per animal, 3-4 tiled regions were captured. Images were analyzed using ImageJ software (Fiji). First each channel underwent background subtraction. Image segmentation was achieved using Cy3 channel to identify αSMA positive vessels. Images were then binarized using the triangle algorithm for thresholding. The binary image was converted to a mask and Cy3+ve areas were eroded to remove small particles, remaining positive areas were then dilated to capture the areas surrounding vessels. The segmented image was used to define measured areas on 488-channel image by redirecting measurements and using the “Analyze Particles” function in ImageJ. Results per image were written to csv files analyzed in R4.5.1.

### Statistics

Continuous physiology and weight data were reported as mean with SD. Edema was quantified by measuring difference in heart-lung block weight after vs. before EVLP. Repeated measurements were compared using 2-way analysis of variance (ANOVA). Static data were compared using one-way ANOVA. Statistical significance was assigned to a p < 0.05. GraphPad Prism Version 10.3.1 was used for all statistical analyses.

## Results

### IGL-2M Prevents Edema Development After Prolonged Static Cold Preservation

**FIGURE 2:**
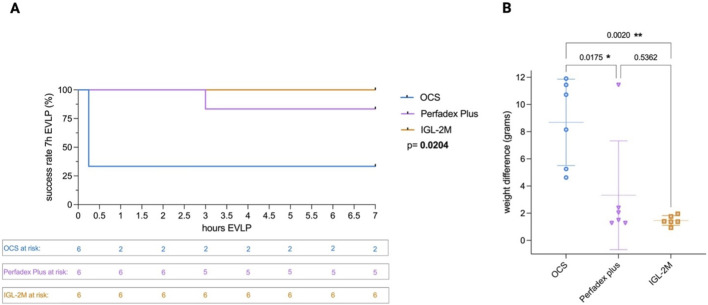
Proportion of experiments developing edema. **(A)** 7 h *ex vivo* lung perfusion success rate. Log-rank test: p < 0.05. **(B)** Lung weight gain after *ex vivo* lung perfusion. Statistical analysis was performed using one-way ANOVA.

All six *IGL-2M* experiments reached 7 h EVLP. In *Perfadex Plus* group, 5/6 experiments reached 7 h EVLP while one experiment failed prematurely after 3 h due to massive edema. In *OCS* group 2/6 reached 7 h EVLP while 4 experiments failed prematurely after 0.25 h due to massive edema (p < 0.05) (Figure 2A).

Weight difference between heart-lung block after vs. before EVLP was taken as indicator of edema development. Weight gain in *IGL-2M* group was lower compared to *OCS* (p < 0.01) and no significant difference was found compared to *Perfadex Plus* (p = 0.54) (Figure 2B).

### IGL-2M Results in Stable Pulmonary Vascular Resistance and Results in Higher Pulmonary Compliance Compared to OCS

**FIGURE 3:**
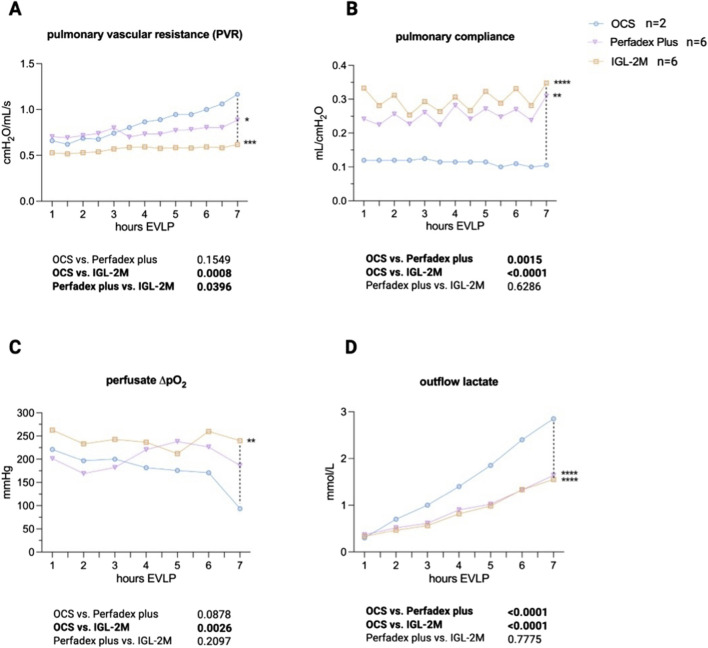
Physiological data during 7 h *ex vivo* lung perfusion. **(A)** Pulmonary vascular resistance. **(B)** Pulmonary compliance. **(C)** Perfusate oxygenation (differential outflow minus inflow partial O2 pressure). **(D)** Outflow lactate levels. Statistical analysis was performed using two-way ANOVA.

After 7 h EVLP, pulmonary vascular resistance remained stable and was lower in *IGL-2M* compared to *OCS*, which showed a worsening trend over time (p < 0.01) and *Perfadex Plus* (p < 0.05) (Figure 3A). Compliance was higher in *IGL-2M* than in *OCS*, which started with a lower value from the beginning (p < 0.01), with no difference compared to *Perfadex Plus* (p = 0.63) (Figure 3B). Pulmonary gas exchange was evaluated using perfusate ΔpO2, showing better oxygenation in *IGL-2M* than in *OCS* (p < 0.01) and no difference compared to *Perfadex Plus* (p = 0.21) (Figure 3C). Cellular damage was assessed by lactate levels in the effluent, with both *IGL-2M* and *Perfadex Plus* having lower lactate levels than *OCS* (p < 0.01) with a trend over time (Figure 3D).

### IGL-2M Results in a Limited Release of Inflammatory Biomarkers in the Perfusate

**FIGURE 4:**
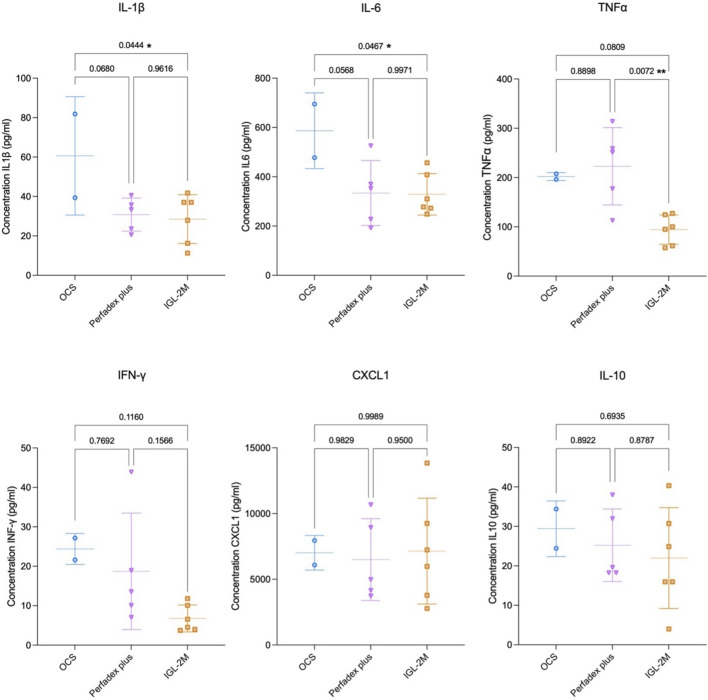
Cytokine expression in the perfusate at 7 h of *ex vivo* lung perfusion. Data are plotted as effective concentrations (pg/mL). Statistical analysis was performed using one-way ANOVA.

Perfusate samples collected after 7 h EVLP in *IGL-2M* showed lower concentrations of pro-inflammatory cytokines IL-1β (p < 0.05) and IL-6 (p < 0.05) compared to *OCS*, and no difference compared to *Perfadex Plus* (p = 0.96; p = 0.99). *IGL-2M* showed the lowest concentration of TNF-α (vs. *OCS* p = 0.08; vs. *Perfadex plus* p < 0.05). Compared to *OCS*, *Perfadex Plus* showed a trend towards lower inflammatory cytokine release (Figure 4).

### IGL-2M Results in a Limited Release of Inflammatory Biomarkers in the Bronchoalveolar Lavage

**FIGURE 5:**
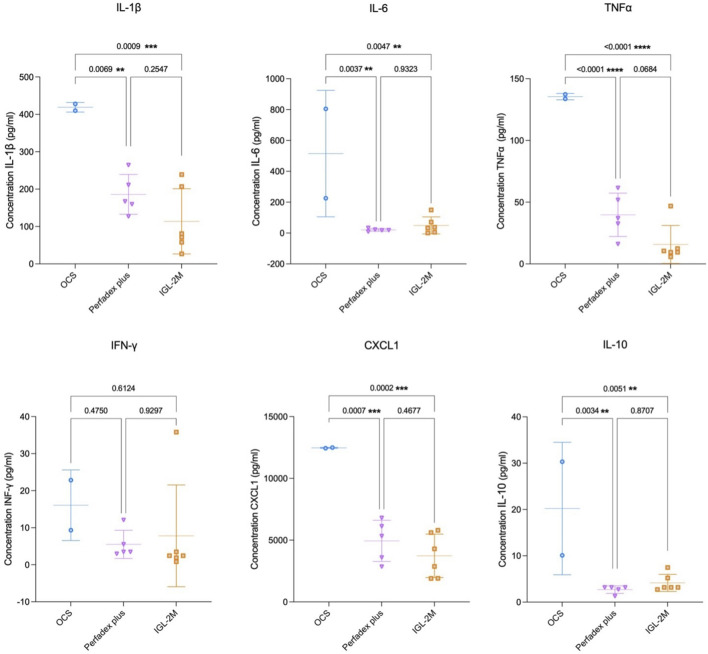
Cytokine expression in the bronchoalveolar lavage samples at 7 h of *ex vivo* lung perfusion. Data are plotted as effective concentrations (pg/mL). Statistical analysis was performed using one-way ANOVA.

BAL samples collected after 7 h EVLP in *IGL-2M* showed reduced release of pro-inflammatory cytokines IL-1β (p < 0.01); IL-6 (p < 0.01); TNF-α (p < 0.01); CXCL1 (p < 0.01) and anti-inflammatory cytokine IL-10 (p = 0.05) compared to *OCS*; and no difference for the same biomarkers compared to *Perfadex Plus*. Compared to *OCS*, *Perfadex Plus* showed reduced release of IL-1β (p < 0.05); IL-6 (p < 0.01); TNF-α (p < 0.01); CXCL1 (p < 0.01) and anti-inflammatory cytokine IL-10 (p < 0.01) (Figure 5).

### IGL-2M and Perfadex Plus Reduce Inflammation in Lung Tissue

**FIGURE 6:**
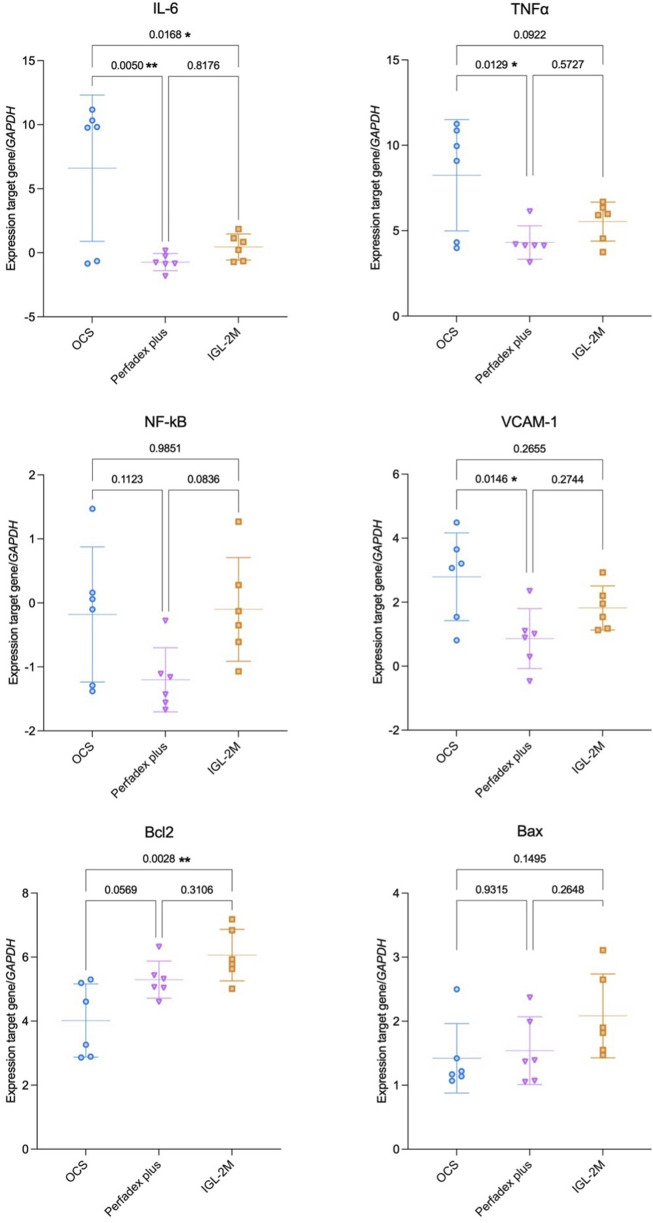
The mRNA expression of cytokines, transcription factor (NF-kB), adhesion molecule (VCAM-1) and proteins involved in the apoptotic signaling in the lung tissue were quantified relative to the housekeeping gene GAPDH after *ex vivo* lung perfusion. Statistical analysis was performed using one-way ANOVA.

Lung tissue analysis with RT-qPCR showed lower IL-6 gene expression (p < 0.05) in *IGL-2M* compared to *OCS*, with no difference compared to *Perfadex Plus* (p = 0.82). Compared to *OCS*, *Perfadex Plus* showed lower gene expression of IL-6 (p < 0.01); TNF-α (p < 0.05); VCAM-1 (p < 0.05) (Figure 6).

### IGL-2M Induces the Expression of Anti-apoptotic Gene

The anti-apoptotic Bcl2 gene expression was higher in *IGL-2M* (vs. OCS, p < 0.01) with no difference compared to *Perfadex Plus* (p = 0.31) (Figure 6).

### Hyaluronan Associated With the Lung Endothelium Is Preserved With IGL-2M

**FIGURE 7:**
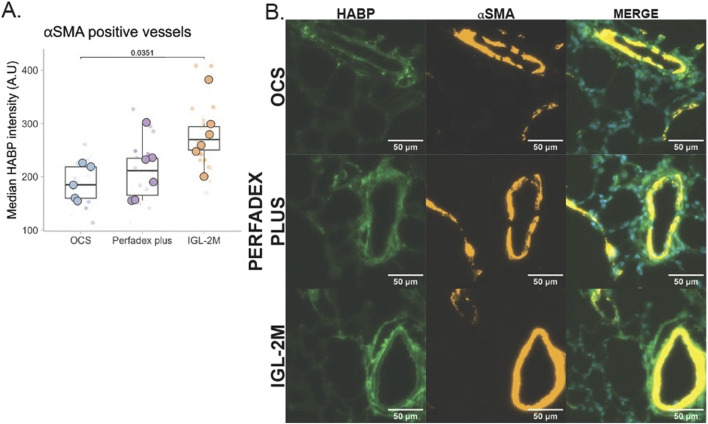
Vascular hyaluronan was quantified using the Hyaluronan Binding Protein (HABP), co-stained with α smooth muscle actin (αSMA), in the rat lung after *ex vivo* lung perfusion. **(A)** Median intensity of HABP signal around αSMA positive vessels. Smaller points represent median intensity values from individual images. Large circles represent the average intensity derived from the individual images obtained per animal. **(B)** representative image of binding protein (green) positive co-localized with αSMA (orange). Statistical analysis was performed using one-way ANOVA.

Hyaluronan detected by biotinylated hyaluronan-binding protein was present in the adventitia of αSMA positive vessels, as well as in the bronchial and bronchiolar epithelium, in the region of the basement membrane. Hyaluronan levels were decreased in αSMA positive vessels across the groups (F (2, 14) = 4.384, *p* < 0.05). Lungs preserved with *OCS* showed reduced vascular hyaluronan levels as compared to *IGL-2M* preserved lungs (Figure 7, *p* < 0.05).

### IGL-2M and Perfadex Plus Preserve Endothelial Glycocalyx Integrity Compared to OCS

**FIGURE 8:**
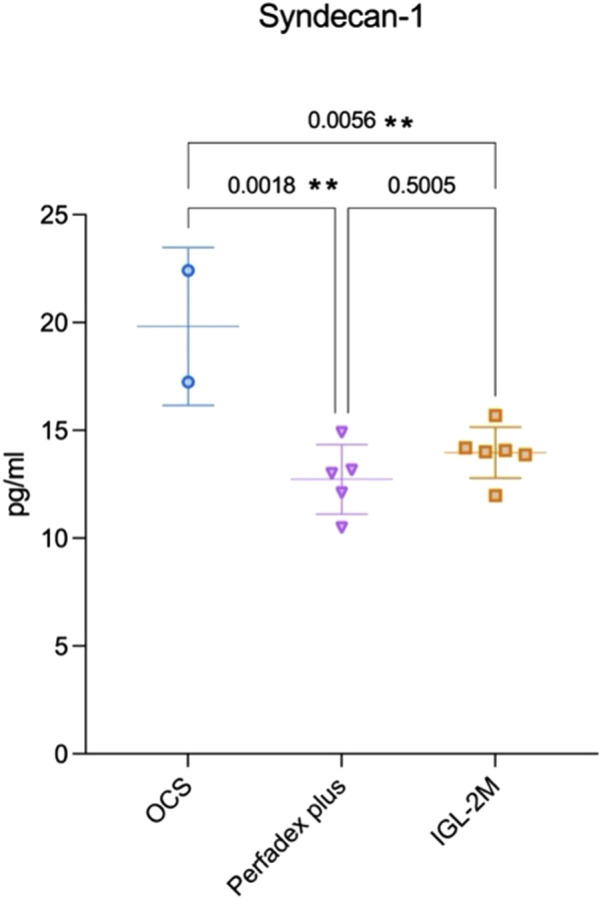
Syndecan-1 expression in the perfusate at 7 h of *ex vivo* lung perfusion. Data are plotted as effective concentrations (pg/mL). Statistical analysis was performed using one-way ANOVA.

Perfusate samples collected after 7 h EVLP in *IGL-2M* and *Perfadex Plus* showed lower concentrations of Syndecan-1 compared to *OCS* (p < 0.05; p < 0.01) (Figure 8).

## Discussion

This rat model of prolonged lung preservation assessed over 7 h EVLP shows for the first time that *IGL-2M*, a PEG-based preservation solution, can be successfully used to flush and preserve rat lungs subjected to prolonged cold ischemia of 18 h. Compared to *OCS*, *IGL-2M* markedly reduced edema formation, preserved pulmonary vascular resistance and compliance, improved oxygenation, reduced lactate release, and attenuated the inflammatory cytokine response. *IGL-2M* was not inferior to *Perfadex Plus*, the current gold standard, and showed an additional effect of TNF-α suppression.

PGD remains a leading cause of early morbidity and mortality after lung transplantation, with an incidence of 30%, largely driven by IRI. While IRI encompasses the cascade of cellular metabolic, inflammatory, and endothelial responses initiated by ischemia and reperfusion, PGD is the clinical manifestation characterized by pulmonary edema, hypoxemia, and impaired compliance [1]. One key feature of IRI is microvascular leakage secondary to endothelial barrier disruption, resulting in uncontrolled transvascular fluid and leukocyte extravasation [4, 18].

Controlled hypothermic storage (CHS) has been introduced in clinical practice, reshaping static lung preservation and allowing prolonged ischemic time [12, 19, 20]. Therefore, minimizing ischemic damage is a key area of investigation. One of the strategies to reduce IRI is to explore new preservation solutions, where the components should aim to prevent the inflammatory processes that typically start during preservation. Minimizing the inflammatory response to ischemia and strengthening endothelial barrier integrity are key therapeutic strategies for reducing IRI and decreasing the incidence of PGD. Our data show that lungs preserved with *IGL-2M* had less edema, lower expression of pro-inflammatory cytokines such as IL-1β, IL-6, TNF-α, and CXCL1, and reduced shedding of syndecan-1, supporting improved preservation of endothelial integrity compared to *OCS*.

*The Perfadex Plus* is the most widely used lung preservation solution [21], with *OCS* lung solution used in the context of portable normothermic perfusion [22]. Both solutions are dextran-based. *Perfadex Plus* is pre-buffered with THAM and supplemented with calcium, which stabilizes endothelial junctions and maintains contractile tone [23]. This likely explains the superior protection compared to *OCS* observed in our model, where lungs preserved with *Perfadex Plus* developed less edema and released lower levels of inflammatory mediators in perfusate, BAL, and tissue. On the other hand, *IGL-2M* replaces dextran with PEG 35kDa, adding anti-inflammatory, antioxidant, and membrane-stabilizing effects [24, 25]. Our data indicate that *IGL-2M* achieves similar preservation compared to *Perfadex Plus*, while showing an additional anti-inflammatory effect through TNF-α suppression.

Physiologically, *IGL-2M*-preserved lungs showed stable and lower pulmonary vascular resistance from the onset of reperfusion, whereas *OCS*-preserved lungs had a progressive rise in resistance during EVLP. Compliance followed a similar pattern, with better compliance in the *IGL-2M* group already at the start of reperfusion. These early differences suggest that lungs preserved with *IGL-2M* experienced less structural and molecular damage during ischemia, allowing for more favorable baseline function when reperfusion began. The vascular staining of HA is likely around veins and venules, both based on structure and previous reports [26]. HA is essential in controlling permeability, but also plays a structural role and affects inflammation [27]. PEG has been shown to stabilize endothelial junctions by promoting VE-cadherin clustering and reducing paracellular gap formation [28]. The protective role of PEG 35 kDa in *IGL-2M* is further supported by *in vitro* studies demonstrating up to a 125% increase in transendothelial electrical resistance (TEER), sustained for over 40 h, compared to the transient effects of other agents. PEG reduces paracellular permeability to FITC-dextran by more than fourfold [28]. Together, these cellular mechanisms likely explain the lack of edema, the immediate physiological stability during EVLP, the decreased vascular leak, lower pulmonary vascular resistance, and reduced inflammatory cytokine release observed in *IGL-2M*-preserved lungs.

*IGL-2M* showed an anti-inflammatory effect at multiple levels. Perfusate analysis demonstrated reduced IL-1β and IL-6 compared to *OCS*, while BAL samples showed lower release of IL-1β, IL-6, TNF-α, and CXCL1. These findings were corroborated by tissue analysis, where IL-6 expression was reduced, and Bcl2 expression increased, indicating anti-apoptotic signaling. Moreover, *IGL-2M* suppressed TNF-α release compared to both *OCS* and *Perfadex Plus*. TNF-α plays a central role in IRI by amplifying endothelial activation, promoting leukocyte adhesion, and driving vascular leak [5]. Its reduction in the *IGL-2M* group points to PEG-mediated modulation of intracellular signaling pathways, beyond colloid effects alone.

Whether this effect will translate into reduced rates of PGD in clinical transplantation remains to be determined.

*Perfadex Plus* also demonstrated clear anti-inflammatory effects compared to *OCS*, lower levels of IL-1β, IL-6, TNF-α, CXCL-1 and IL-10, consistent with its calcium supplementation. Calcium is essential for preserving endothelial and epithelial integrity by stabilizing adherens junctions. *Perfadex Plus* contains a modified dextran formulation with a lower viscosity and carefully balanced electrolyte/osmolarity profile, reducing the risk of vascular leak. This likely accounts for the lower edema and reduced cytokine release observed in the *Perfadex Plus* group. Thus, while both solutions limit inflammation and preserve function, they probably act through different mechanisms: calcium-mediated stabilization for *Perfadex Plus* and PEG-mediated for *IGL-2M*.

Our findings are consistent with prior studies in liver transplantation, where PEG-containing solutions have been shown to preserve mitochondrial function, redox balance, and endothelial glycocalyx integrity more effectively than traditional solutions [25, 29–32]. PEG has also demonstrated anti-inflammatory, immunosuppressive, and cell-membrane-stabilization effects in the setting of intestinal IRI, increasing with higher dose [33–36]. The reproducibility of PEG’s protective effects across organs suggests the feasibility of a universal preservation solution. A single PEG-based formulation for multiple organs could streamline procurement, simplify logistics, and expand the use of marginal donors.

This study has several limitations. Firstly, the high failure rate in the *OCS* group reduced the number of evaluable data points at the 7 h EVLP time point to only n = 2. This affects the statistical power, so the 7 h comparison should be interpreted with caution. However, this reflects the inability of *OCS*-preserved lungs to withstand prolonged cold ischemia followed by EVLP, resulting in edema and endothelial injury, which serve as important biological indicators of inferior preservation capacity under extended cold ischemia, rather than a technical failure. Secondly, 18 h of cold ischemia may exceed the tolerance of the *OCS*, potentially leading to early EVLP failure. However, this observation is indicative of solution-specific performance rather than a real limitation inherent to the study design. Third, direct visualization of endothelial glycocalyx integrity was not possible due to the experimental setup, as HA is only one component of the glycocalyx. Nonetheless, an ongoing transmission electron microscopy study will offer further evidence. Fourth, since this is a rodent model, caution is needed when translating findings to human lungs. Fifth, mechanistic studies were limited by the limitation of obtaining sequential tissue samples during ischemia and reperfusion. Nevertheless, the robust physiological, biochemical, and inflammatory data provide consistent evidence supporting the protective role of *IGL-2M*.

## Conclusion

*IGL-2M* preserved lung function during prolonged cold ischemia at least as effectively as *Perfadex plus*, with additional anti-inflammatory benefits on TNF-α. Compared with *OCS*, *IGL-2M* limited edema formation and improved physiological stability. These findings justify further evaluation of *IGL-2M* in large animal models and provide a strong rationale for clinical translation, with the potential to establish a single PEG-based fluid as a universal organ preservation solution.

## Data Availability

The original contributions presented in the study are included in the article/supplementary material, further inquiries can be directed to the corresponding author.
